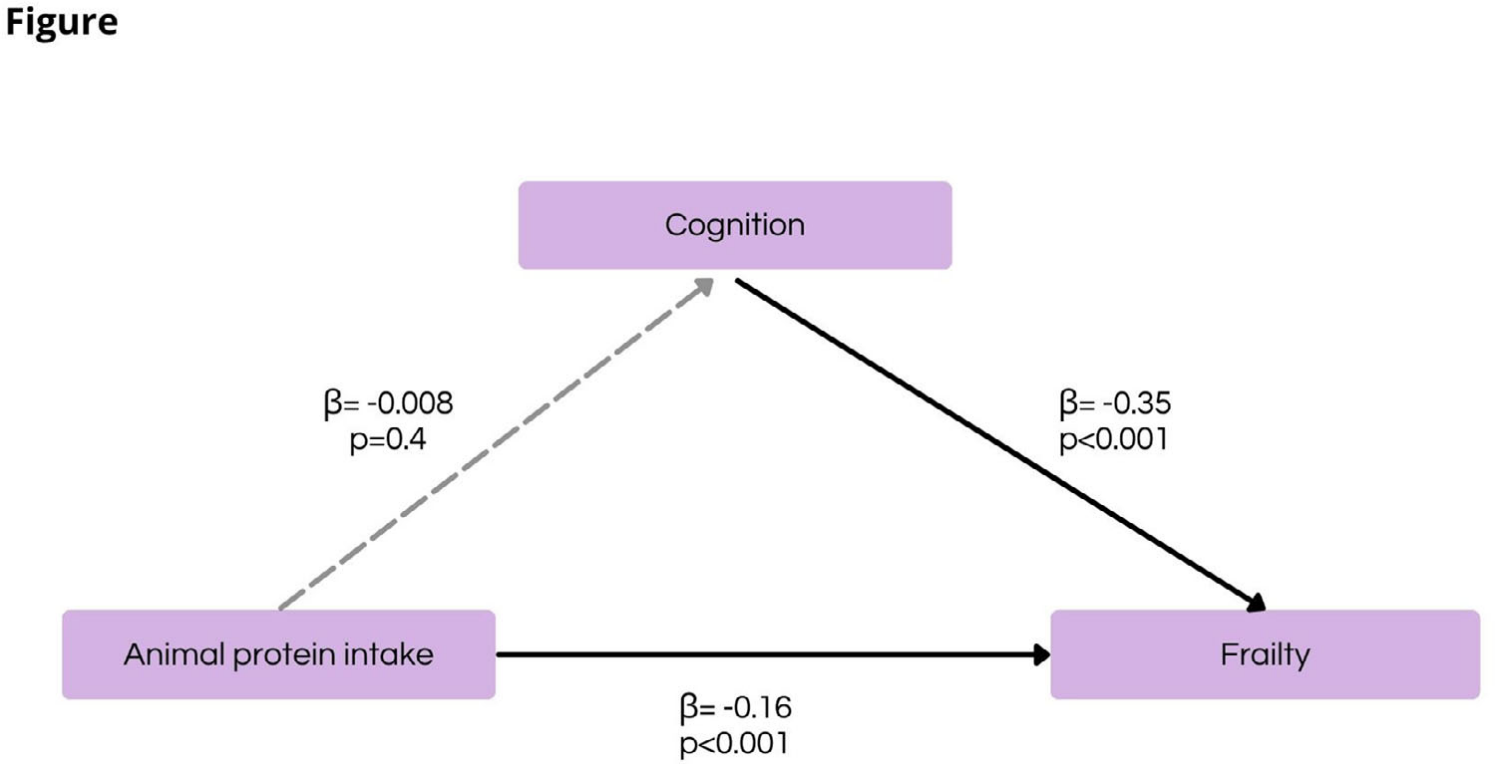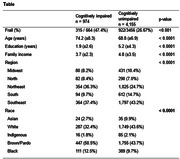# Animal protein intake is associated with frailty but not with cognition: an ELSI‐Brazil preliminary study of food consumption

**DOI:** 10.1002/alz.092777

**Published:** 2025-01-09

**Authors:** Vivian Silveira Vasques, Wyllians Vendramini Borelli, João Pedro Ferrari‐Souza, Núbia Alencar de Freitas, Patricia Pauli, Claudia Kimie Suemoto, Eduardo R. Zimmer

**Affiliations:** ^1^ Universidade Federal do Rio Grande do Sul, Porto Alegre, Rio Grande do Sul Brazil; ^2^ Federal University of Rio Grande do Sul, Porto Alegre, Rio Grande do Sul Brazil; ^3^ Memory Center, Hospital Moinhos de Vento, Porto Alegre, RS Brazil; ^4^ Universidade Federal do Rio Grande do Sul, Porto Alegre Brazil; ^5^ Division of Geriatrics, University of São Paulo Medical School, São Paulo Brazil

## Abstract

**Background:**

Diet is considered a complex modifiable risk factor for dementia and frailty. Some dietary patterns such as the MIND diet have been associated with a reduced risk of cognitive decline and dementia. Studies have shown mixed results with protein intake and frailty. However, the association of animal protein intake with cognitive performance and frailty is yet unclear. This study aimed to investigate the association of food group consumption with cognition and frailty in Brazilian older adults.

**Method:**

This cross‐sectional study included baseline data from the Brazilian Longitudinal Study of Ageing (ELSI‐Brazil). The sample comprises cognitively unimpaired (CU, n = 4.155) and cognitively impaired (CI, n = 974) older adults (Table). Frailty was defined by weight loss, weakness, slow walking speed, exhaustion, and low physical activity. Cognition was calculated with a z‐score of orientation scores, animal fluency and memory recall. Individuals were defined as CI if the global cognition score was below 1SD of the sample mean. The association between food consumption and cognition was evaluated using a linear regression model corrected for age, sex, education, BMI, income, and frailty. A mediation model was developed with the association between cognition, frailty and animal protein intake (Figure).

**Result:**

CI individuals showed increased rates of frailty (47.4% vs. 26.6%, p<0.001), and presented decreased weekly intake of fruits (8.9±6.2 vs. 9.6±6.3, p = 0.03) when compared with CU. Weekly intake of vegetables and animal protein was similar between groups (Table). Linear regression model identified total weekly intake of animal protein was associated with frailty, but not with cognition (Figure). Consuming animal protein in less than 5 days per week was associated with increased cognitive scores (β = 0.24±0.08, p<0.001), but this association was not significant when analyzing subgroups of animal protein (red meat, fish or chicken).

**Conclusion:**

Our results suggest that weekly animal protein intake is associated with frailty but not cognition. However, intake of animal protein less than 5 days a week may have a neuroprotective effect. Further studies should understand the complex interaction of animal protein consumption in cognition and frailty.